# Effects of a cognitive ergonomics workplace intervention (CogErg) on cognitive strain and well-being: a cluster-randomized controlled trial. A study protocol

**DOI:** 10.1186/s40359-019-0349-1

**Published:** 2020-01-02

**Authors:** Virpi Kalakoski, Sanna Selinheimo, Teppo Valtonen, Jarno Turunen, Sari Käpykangas, Hilkka Ylisassi, Pauliina Toivio, Heli Järnefelt, Heli Hannonen, Teemu Paajanen

**Affiliations:** Finnish Institute of Occupational Health, P.O Box 40, 00032 Helsinki, Finland

**Keywords:** Working conditions, Cognitive strain, Disruptions, Interruptions, Information overload, Workplace intervention, Cognitive ergonomics, Recovery

## Abstract

**Background:**

Cognitively straining conditions such as disruptions, interruptions, and information overload are related to impaired task performance and diminished well-being at work. It is therefore essential that we reduce their harmful consequences to individual employees and organizations. Our intervention study implements practices for managing the cognitive strain typical to office work tasks and working conditions in offices. We will examine the effects of a cognitive ergonomics intervention on working conditions, workflow, well-being, and productivity.

**Methods/design:**

The study is a stratified cluster randomized trial. The clusters are work units, for example, teams or offices. The four participating organizations entered a total of 36 clusters, and we invited all 1169 knowledge employees of these units to participate. We randomly allocated the clusters into an intervention group (cognitive ergonomics) or an active control group (recovery supporting). We invited an additional 471 participants to join a passive control group only for baseline and follow-up measurements, with no intervention.

The study consists of a baseline survey and interviews and observations at the workplace, followed by an intervention. It starts with a workshop defining the specific actions for the intervention implementation stage, during which we send task reminder questionnaires to all employees to support behaviour change at the individual and team levels. The primary outcome measure is perceived frequency of cognitive strain from working conditions; the secondary outcome measures include subjective cognitive load, well-being, workflow/productivity, and cognitive stress symptoms. Process evaluation uses the quantitative and qualitative data obtained during the implementation and evaluation phases. The baseline measurements, intervention phase, and end-of-treatment measurements are now complete, and follow-up will continue until November 2019.

**Discussion:**

There is a need to expand the research of cognitive strain, which poses a considerable risk to work performance and employee well-being in cognitively demanding tasks. Our study will provide new information about factors that contribute to such strain. Most importantly, the results will show which evidence-based cognitive ergonomic practices support work performance in knowledge work, and the project will provide concrete examples of how to improve at work.

**Trial registration:**

ClinicalTrials.gov, NCT03573674. Registered 29 June 2018.

## Introduction

In modern digitalized work environments, the performance of work tasks relies heavily on cognitive functioning, that is, the mental processes that are involved in information processing such as attention, working memory, decision-making, and learning. These demands are notable in knowledge work jobs that require working with abstract knowledge and acquiring, creating and applying knowledge, as well as continuous on-the-job learning [1, 2]. Cognitive load is caused by the cognitive demands of work tasks, which easily exceed the natural limitations of human cognitive capacities, but strain may also be further increased by working conditions. Many working conditions impair cognitive performance, as research into disruptions and interruptions has demonstrated [3, 4]. These conditions, as well as requirements related to the fragmentation of work, multitasking and information overload have proven to be typical straining features in many fields, including knowledge work tasks [5–7].

Cognitive strain related to work demands or working conditions is a notable risk factor for work performance, as it directly affects the human ability to master cognitively demanding work tasks. Disruptions in the work environment, such as speech and office noise disrupt office-related tasks (e.g. [4, 8, 9]) and interruptions have harmful consequences for task performance [3, 10, 11]. Furthermore, information overload manifested as multitasking or through new interaction technologies hinder task performance [12–15].

Not only do cognitively straining working conditions directly impair cognitive functioning and task performance; they can also lead to cognitive failures that affect overall performance. In health care, task-related stressors including interruptions and performance constraints, such as obsolete information, have predicted a higher level of attention failure [16, 17], which mediates the influence of workflow interruptions on near-accidents [18].

Previous research thus shows that cognitively straining conditions can have direct effects on task performance, as well as indirect, extensive effects on work performance and productivity if they expose employees to cognitive failure and impair occupational safety and health [3–18]. Research also shows that good work performance and high employee well-being are mutually connected (for a review see [19]). Job satisfaction and well-being at work are associated with better workplace performance [20–22], whereas poor working conditions increase job dissatisfaction, which in turn increases sickness absence [22] and intentions to quit [23]. To sum up, good working conditions, high work performance, and high employee well-being support each other.

It is therefore essential to manage cognitively straining conditions and reduce their harmful consequences for individual employees, teams, organizations, and society. Our approach is to directly affect the conditions that are likely to disrupt human cognitive functioning, thus directly affecting task performance. This approach is related to human factors and job crafting studies which manage conditions and ways of working to support the human capacity to perform well [24–26] and differs from studies that focus on supporting and improving well-being and psychological health at work (for reviews see e.g. [27, 28]). Our focus is on implementing practices that improve work performance and are not harmful, but possibly beneficial to well-being. However, our study design also includes an active control group with a recovery support intervention. Thus, it also contributes to recent research that has combined the improvement of performance and well-being at work [19].

In sum, our study has developed a cognitive ergonomics intervention that aims to reduce cognitive strain at work. Our main research questions are: Does the intervention influence the frequency of cognitively straining conditions, and does it result in improved workflow, performance, and well-being?

### Improving human performance and well-being with ergonomics

Our workplace cognitive ergonomics intervention focuses on ergonomic (or human factors) practices that aim to ensure ‘appropriate interaction between work, product and environment, and human needs, capabilities and limitations’, as defined by the Ergonomics and Human Factors Society [29]. In cognitive ergonomics, the focus is on human cognitive functioning and the conditions affecting this, and on making human-system interaction at work compatible with human cognitive abilities and limitations [29, 30].

In our intervention study, we define cognitive ergonomics in the context of office work and focus on the factors reducing the cognitive strain related to working conditions. As our study focuses on knowledge-work office environments, it covers a type of work that concerns a large portion of the workforce. This group is still underrepresented in cognitive ergonomics (or human factors) studies that focus on cognitively intensive aspects of work. Moreover, previous results concerning cognitive functioning in safety-critical or high-demand domains such as health care, aviation, or the nuclear industry, with highly trained groups of employees cannot be directly generalized to the cognitively demanding work of more common work places. In contrast, our study specifically targets the general population in offices and broadens our understanding of both their cognitively intensive tasks and their cognitively demanding working conditions.

Previous studies on job crafting and human factors provide examples of interventions that have focused on the cognitive ergonomic themes relevant to our intervention, that is reducing disruptions, interruptions, and information overload. For example, quiet hours free of any phone calls, visitors, or incoming emails aim to reduce disruptions and interruptions and to enable the employee to focus on the task at hand; results show improvement in performance during the quiet hour, but also in overall day-level performance [31], for contrasting results, see [32, 33]. Furthermore, better practices for handling new emails can reduce information overload and stress, for example, checking emails three times rather than unlimited times a day leads to lower daily stress which predicts better perceived productivity [34]. Moreover, collecting several questions and asking them at the same time rather than constantly interrupting increases the efficiency of knowledge sharing and helping between employees [33].

Some intervention studies have also focused on improving well-being in knowledge work jobs but have included cognitive ergonomic actions at the workplace. For example, initiatives have been related to dealing with non-essential interruptions, reducing interruptions, setting aside thinking time, creating open office rules, and having do-not-disturb signs [2], but the interventions have not differentiated the effects of these cognitive ergonomic actions from those of other actions. Although the study by Sørensen and Holman [2] highlighted several cognitive ergonomics aspects, their intervention programme aimed at improving well-being. In contrast, our main interest is in improving cognitive ergonomics at work and in evaluating how it affects performance and productivity. Evaluating productivity in knowledge work remains a challenge as the concrete measures of productivity that are relevant in, for example, physical and safety critical work environments, such as levels of sickness absence or occupational accidents, are relatively low in office work. There is a lack of comparable objective measures of productivity in the research literature and in knowledge work in general, but our study will increase the sparse research knowledge on this topic.

In summary, more research is needed to recognize cognitive strain in knowledge work in offices, to identify actions that improve cognitively straining working conditions, and to understand how improvement of cognitive ergonomics at the workplace affects workflow, productivity, and well-being at work.

We have developed an intervention program, the Cognitive Ergonomics Intervention at the Workplace (CogErg), which combines three essential cognitive ergonomic themes: disruptions, interruptions, and information overload, themes that are likely to transfer to many organizational contexts. Since several organizational factors may affect which cognitive ergonomic improvements are possible, we do not focus on a single predefined specific condition or action, as is the case with many previous interventions. In the intervention kick-off workshop, a group of participating workers discuss the three themes and co-develop cognitively sound work practices compatible with the organizational context. Based on interviews and the workshops, specific work practices and concrete actions are defined, and these are communicated to workers during the intervention implementation phase. Our intervention programme thus expands existing programs by combining three large cognitive ergonomics themes essential to knowledge work in offices. Moreover, the specific and concrete actions under these general themes are adapted and tailored to the organizational context.

Furthermore, we recognize that conditions in work life are complex and that the amounts of disruptions, interruptions, information overload, and working together often go hand in hand. Therefore, a truly effective intervention is likely to require joint effort rather than the actions of individual employees. In our CogErg intervention, it is emphasized during implementation that change requires concerted action: in the intervention task reminder questionnaires we ask employees to share and discuss the themes and actions with their teammates and supervisors. Our study thus expands existing workplace intervention programs by not only including several relevant cognitive ergonomic themes and adapting the actions to the context, but also by implementing a complex workplace intervention on a group level rather than focusing on individuals. Thus, we contribute to a growing field of group-level workplace intervention studies and extend it to cognitive ergonomic themes. We also evaluate the process of implementation [35] to better understand the mechanisms of the intervention and how the context affects and moderates the effects on the outcome variables [36–38].

### Aims and hypotheses

In this paper, we present in detail our developed and implemented cognitive ergonomics workplace intervention programme, as well as the design and methods of our study.

The first objective of the study is to implement cognitive ergonomic improvement actions that aim to reduce the level of cognitively straining working conditions, that is, disruptions, interruptions, and information overload at workplace. These conditions are typical in many fields and notable risks for work performance [3–15]. Previous results also suggest that it is possible to change these conditions, which may have positive effects on cognitive load and its consequences, but the research evidence is not yet convincing [2, 31, 33, 34]. Our first research question thus is whether a cognitive ergonomics intervention programme focusing on disruptions, interruptions, and information overload is effective and whether it affects the primary outcome measures related to cognitively straining working conditions. Our primary hypothesis is:

1. The cognitive ergonomics intervention will decrease the reported level of disruptions, interruptions, and/or information overload, in comparison to an active control group and a passive control group.

The second objective is to study whether the cognitive ergonomics intervention and its effects on working conditions are also revealed in the secondary outcome measures related to the subjective measures of workflow, productivity, cognitive strain, and well-being, in comparison to the results of an active control group (recovery support). These outcome measures are core elements of productive and healthy work life, and previous research shows that cognitively loading conditions may underlie related problems; moreover, working conditions, work performance and employee well-being are mutually connected [16–23]. Our second and third research questions are thus whether a cognitive ergonomics intervention programme for decreasing the level of cognitively straining working conditions also affects performance and well-being. The secondary hypotheses are:

2. The cognitive ergonomics intervention will improve workflow and productivity and decrease cognitive strain, in comparison to an active control group and a passive control group
3. The cognitive ergonomics and recovery support interventions will improve well-being in comparison to a passive control group

Furthermore, we also aim to understand the process of the cognitive ergonomics intervention: what components related to intervention implementation and organizational context may moderate the effects, what factors may hinder or promote the effects of the intervention, and what change mechanisms may underlie behaviour change and organizational learning when the level of cognitive ergonomics is improved [35]. We will thus analyse the key components suggested in the guidelines for process evaluation of complex interventions: context, implementation, and the mechanisms of impact [35] and we will use the mainly qualitative information that will be obtained during the intervention phase when interpreting the intervention outcomes. We will explore:

4. How do the factors related to the implementation of the intervention (e.g. the reach of the intervention) moderate the effects of the intervention; are the effects larger in the groups with a larger participation percentage?
5. How do the factors related to the organizational context (e.g. the commitment of the organization and supervisors) moderate the effects of the intervention; are the effects larger in the groups with more active supervisors?
6. How do the factors related to the mechanisms of impact (e.g. participant responses to the intervention) moderate the effects of the intervention; are the effects larger in the groups that report concrete behaviour change?

## Methods/design

The study began in August 2017 with participant recruitment and the intensive intervention phase ended in December 2018. The final follow-up measurements are estimated to end in November 2019. Results are expected in 2020. This study has been registered with the ClinicalTrials.gov registry (NCT03573674).

### Study monitoring

The research trial procedures are audited by the steering group twice a year. The steering group monitors and evaluates data management and, if necessary, requests changes to the protocol. The principal investigator (VK) has access to all data and results and will discuss with the steering group if changes in the protocol or a need to terminate the trial would appear. If the protocol is modified, the revision is submitted to the ethics committee for approval before implementation, and the ClinicalTrials.gov registry is informed. The principal investigator (VK) reports incidents related to data management and changes in the project plan, if any, to the FIOH Data Protection Officer and the steering group and the funder, respectively. A separate data-monitoring committee was not considered necessary as the risks to participants were expected to be minimal.

### Study design

**Fig. 1:**
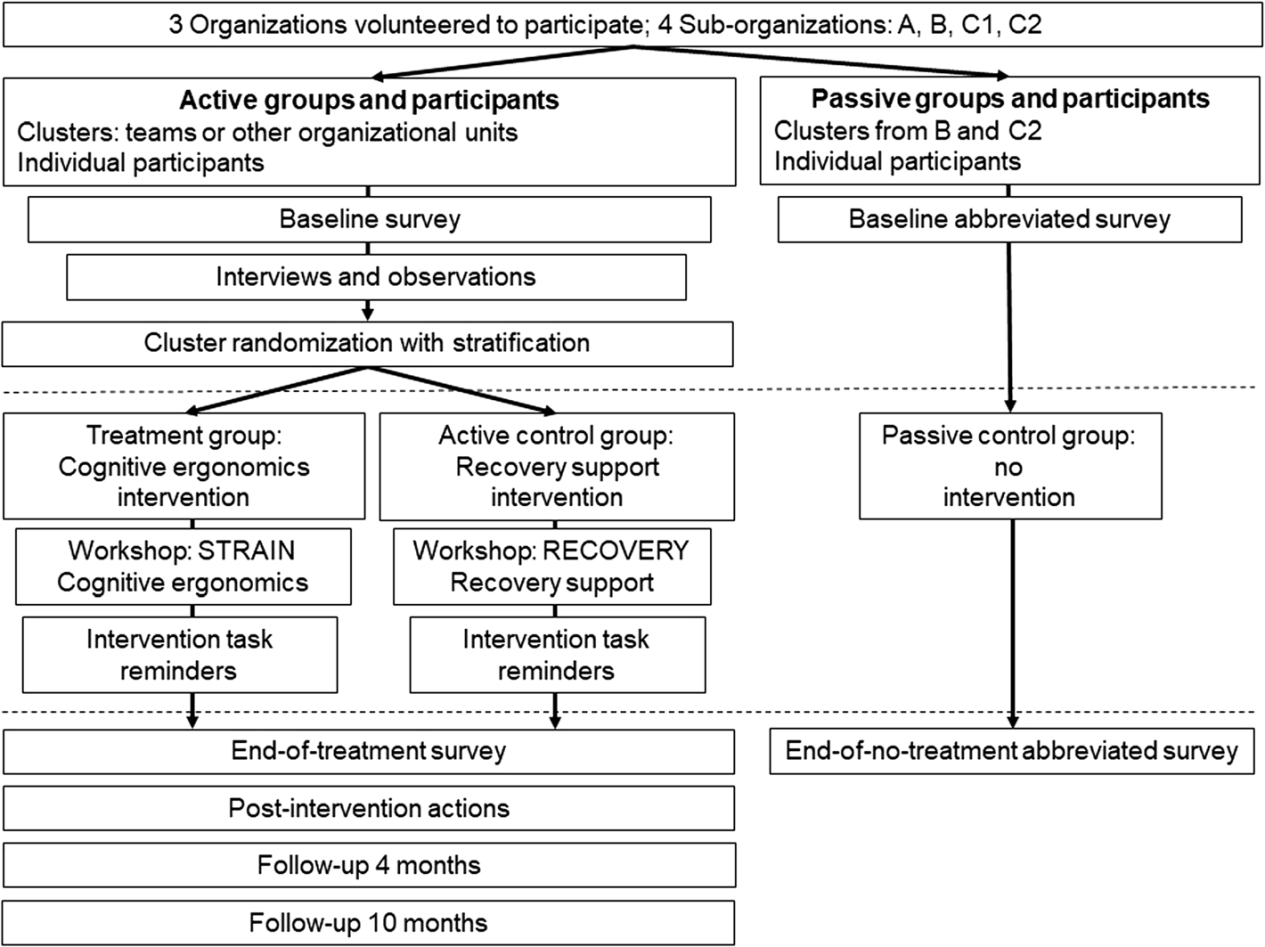
The flow chart of the Cognitive ergonomics intervention study CogErg

The study design is a randomized controlled trial (RCT), applying cluster randomization with stratification allocated groups and parallel assignment. Primary stratification was by organization. Each organization’s clusters were randomly assigned to treatment (Cognitive ergonomics intervention, CE) and active control (Recovery support intervention, RS) conditions. There were four measurement points (Fig. 1): Baseline (before the intervention), End-of-treatment (after the intervention), Follow-up at 4 months, and Follow-up at 10 months. Both in the treatment and the active control interventions the stages consisted of two parts: a kick-off workshop and following weekly intervention task reminders (10 for all participants and an additional 3 for supervisors) which were delivered between the Baseline and End-of-treatment measurements points (Tables 1 and 2). Two organizations also provided clusters for a non-randomized passive control group (PC), which received only the baseline and end-of-treatment surveys.

**Table 1:** Themes of intervention task reminder questionnaires for supervisors (SQ1–3) and employees (TQ1–10). The structure of each questionnaire is the same for the treatment (Cognitive ergonomics intervention, CE) and active control (Recovery support intervention, RS) groups; only the content differs. Each task questionnaire includes an invitation and information on the study and presents the key issue of the reminder, that is, what themes the team should discuss

Questionnaire theme	SQ1	TQ1	TQ2	TQ3	TQ4	TQ5	SQ2	TQ6	TQ7	TQ8	TQ9	TQ10	SQ3
Practices for implementing ground rules; factors that support or prevent implementation (1 MCQ/ 2 OQ)	x						x						
Level of ground-rule implementation (1 MCQ/ 2OQ)	x						x						x
Which working methods and ground rules are implemented; factors promoting and preventing implementation (3 OQ)							x						
Number of tasks and how well the unit completed them (2 MCQ/ 1 OQ)													x
Invitation to workshop, evaluation of personal value of managing CE or RS (6MCQ)		x											
Evaluation of self-perceived stress and recovery (2 MCQ)		x						x				x	
Evaluation of task completion (2 MCQ)		x	x	x	x	x		x	x	x	x	x	
Determining goals to improve workflow (group discussion) (1OQ)		x											
Presentation of units’ common working methods and ground rules (2 MCQ/ 2 OQ)			x										
Naming working methods and ground rules that both single employees and their units decided to follow				x	x	x			x	x	x		
Frequency of following ground rules related to content*; factors that support or prevent achievement of goals (1 MCQ/ 2 OQ)				x					x				
Frequency of following ground rule-related content**; factors that support or prevent achievement of goals (1 MCQ/ 2 OQ)					x					x			
Frequency of following ground-rule content***; factors that support or prevent achievement of goals (1 MCQ/ 2OQ)						x					x		
How implementation of ground rules is going; factors that support or prevent achievement of goals (3 MCQ/ 3OQ)								x					
Group- and individual-level implementation of new working methods (2 MCQ/ 2OQ)												x	

*MCQ* multiple choice question, *OQ* optional question, writing down other rules that unit have agreed on and other comments and thoughts *Content for CE: Reducing distractions and for RS: Managing strain; **Content for CE: controlling interruptions and for RS: Promoting recovery; ***Content for CE: managing information overload and for RS: Improving work/life balance

**Table 2:** Description of outcome measures assessed by self-report questionnaires*

Outcome	Description
Primary	
Cognitive Strain Prevalence	An average of the frequency of cognitive strain over the three subscales (presented below): Disruptions, Interruptions, and Information overload items in the BWP and KWCFS questionnaire modules.
Disruptions (subscale)	Two items from the BWP: working in a noisy environment and working in an environment with distracting objects.
Interruptions (subscale)	One item from the BWP and two items from the KWCFS: i) dealing with interruptions at work, ii) switching from one set of tasks to another before finishing the first, and iii) switching to another task that interrupts the ongoing task.
Information Overload (subscale)	Seven items from the BWP and three items from KWCFS: recalling detailed information, recalling the exact order of work phases, remembering agreed appointments, constantly switching attention from one thing to another, using several different devices, monitoring several things and observing changes, working according to contradictory instructions, having too many messages to handle, not knowing whom instructions concern, and not understanding instructions.
Secondary	
Cognitive Strain	An average of cognitive strain over the three subscales (Disruption, Interruptions, Information overload). First, based on the ten BWP items, the values of the engagement–strain assessments weighted by the frequency value of the item. Second, based on all the 39 BWP items, the values of the engagement–strain assessments weighted by the frequency value of the item.
Well-being	Several independent items or scales. Stress as a single item assessing the experience of stress on a scale of 0 to 10. Recovery as a single item assessing the ability to recover after work on a scale of 0 to 10. General Health as a single item from the COPSOQ-II questionnaire. Burnout with the four-item COPSOQ-II Burn Out index.
Cognitive Stress Symptoms	The four-item CS scale from the COPSOQ-II questionnaire.
Work Flow / Productivity	Several independent scales. Presenteeism and Subjective Productivity: two items from the Health and Work Performance questionnaire (B14 and B15). Memory Failures: an average of five items from the KWCFS (the frequency of memory failures at work, e.g. inability to remember a password). Attention Failures: an average of five items from the KWCFS (the frequency of attention failures at work, e.g. inability to stay focused on work tasks). Multitasking Failures: an average of three items from the KWCFS (the frequency of the aspects of multitasking failures at work, e.g. trying to do too many tasks at once.)

*Questionnaires are presented in Table 3. *KWCFS* Knowledge Work Cognitive Failure Scale, *BWP* Brain Work Profile, *COPSOQ-II* Copenhagen Psychosocial Questionnaire II

### Study sample and recruitment

The participants are employees of given teams or groups in three organizations that had committed themselves to the study by January 2017. The participating organizations were recruited during 2016–2017 by contacting different organizations that employed knowledge employees, such as office employees and experts whose work tasks are cognitively rather than physically demanding. These organizations include two large divisions of one public organization, and two private enterprises in Finland, which were considered four organizations in the stratification. The organizations’ headquarters are located in the metropolitan area, but they all have regional offices in several other parts of Finland. The organizations represent different fields of business and trade, such as planning, consulting, marketing, customer service, administration, and civil service, in the context of the construction industry, transportation, and government. The participating teams of these organizations mainly work in open-plan offices and the teams may be distributed over several urban areas in Finland.

The recruitment of employees began in October 2017, when the participating organizations were asked to share general information to their teams, employees, and supervisors. The researchers participated in the supervisors’ meetings to briefly provide general information and discuss the study. The employees of the teams were separately recruited for each study phase via email. The longitudinal sample design is fixed panel plus new employees, that is, new employees are added to the original sample during the different stages of the study, and in practice, the employees who no longer work in the organization or the participating unit are excluded from the updated participant lists and dropped from the panel. Participants were recruited during the time slots agreed on with their organization. The contact information of all the members of the participating teams was signed over to the research group before the onset of the first phase. To support retention, we provide participants written feedback on all study questionnaires. In addition, supervisors are contacted regularly during the intervention phase to maintain participation rates. However, participants may withdraw from the study for any reason at any time.

#### Baseline survey, interview, and observation recruitment

We recruited the participants for the baseline survey in November 2017 to February 2018 by sending them a personal email invitation to participate in the study, with a link to the study information and baseline survey. We recruited the participants for the interviews and observations (only active groups) by asking baseline survey participants to volunteer for interviews and observations. We selected the participants to contact so that the work tasks and teams of the organizations were equally represented. We contacted them directly by sending an invitation to participate to 1–3 employees in each clustered team who represented different work roles, gender, and number of years working in the organization. We continued to recruit participants until we obtained the required number.

#### Workshop and intervention implementation recruitment

We recruited the workshop participants at the initial stage (active intervention groups) of the intervention from February 2018, through the organizations’ contact persons, who made the practical arrangements and invited the participants. In two organizations, all the employees from the participating teams were invited, but two other organizations decided to invite mainly supervisors and/or active key figures from among team members. We invited the intervention task reminder questionnaire participants at the implementation stage (active groups) of the intervention from the population invited to the baseline survey plus new members of the participating teams (a fixed panel plus new employees longitudinal design). We also invited the supervisor task questionnaire participants during the intervention implementation, from the participating team supervisor lists.

#### End-of-treatment, post-intervention and follow-up recruitment

We recruited the participants for the end-of treatment surveys from the updated participant lists which included all current employees (active and passive groups) in the participating teams of the participating organization. The participants in the post-intervention actions include those of the evaluation workshops; 3–10 employees or supervisors who have actively participated will be invited along with 3–10 key members from human resources, occupational health and safety, and the management units. Other post-intervention actions that may involve the research group include project-related knowledge-sharing actions.

We will recruit the participants for the four- and ten-month follow-up surveys from the updated participant lists (active groups), which includes all the current employees in the participating teams of the participating organizations.

### Inclusion criteria

The participating organizations selected the units, that is, teams or other organizational units (clusters) to be included in the study. The inclusion criteria were: a) units must include knowledge employees, such as office employees and experts whose work tasks are cognitively rather than physically demanding, b) work performance requires learning and updating knowledge and skills, and c) work is highly dependent on information and communication technology. Furthermore, each participating organization was required to offer several units/teams for the study, to enable us to conduct stratified randomization.

### Sample size

We assumed that all employees would meet the inclusion criteria, and that at least 60% of the participants invited to the baseline survey would respond. It was further assumed that the follow-up attrition would be less than 50%. The meaningful significant difference estimation was calculated on the basis of sub-group differences in previously collected unrelated data (*N* = 2154) on cognitively straining working conditions. The sample size estimations were based on the average size of the teams; we could not define a fixed group size as the clusters in this study are real organizational units that vary in size. According to the power analyses, 32 groups with 15 eligible participants in each is the estimated sample size required to achieve a statistical power of 0.95 with an alpha of 0.05, if the ICC (intraclass correlation coefficient) is assumed to be 0.3 (For detailed information on how group size and ICC affect power, see [43]). Thus, to achieve a significance level of 0.05 when requiring 0.95 power, a sample size of *N* = 480 participants (15 per group) is needed to enable finding a significant difference in the primary outcome of the different arms. A follow-up attrition of 50% and a baseline response rate of 60% would require inviting altogether 1600 participants in order to meet the power requirements defined above. If power was set to 0.80, the required sample size would be 330.

### Randomization

Participants were divided into clusters using existing work communities such as teams, or a shared office. We randomized the clusters into treatment (cognitive ergonomics intervention) and active control (recovery support) groups. Within-organization stratification took into account the type of work tasks within a cluster, the number of respondents in the cluster, and the response rate of the cluster of employees in the first survey. In one organization over 50% of the team members were geographically scattered in some clusters (teams); this factor was therefore used as an additional stratification in this organization. The clusters were ordered using the stratification factors, and a computer-generated random number was used to define whether the even- or the odd-numbered clusters were allocated to the treatment. The allocation ratio was thus 1:1, allocation was not concealed. One member of the research group (PT) planned the randomization procedure which was conducted by PT (for three strata) and VK (for one stratum). The principal investigator (VK) informed the organizations of team allocations, and the contact persons in the organizations invited the teams to the intervention workshops.

### Participants

The total sample in the randomized treatment and active control groups consisted of 1271 office employees from 36 clusters, in four strata (organization A: *n* = 657, B: *n* = 216, C1: *n* = 238, and C2: *n* = 160). We also invited 470 participants from two strata where additional clusters were available (B and C1) to the passive non-randomized control group. In the baseline measurement phase, we received a total of 638 valid responses from the treatment and active control groups (organization A: *n* = 240, B: *n* = 144 and C1: *n* = 153 and C2: *n* = 101), corresponding to a response rate of 50.2% (organization A: 36.5%, B: 66.7% and C1: 64.3% and C2: 63.1%). We obtained 289 valid responses from the non-randomized passive group, the response rate thus being 61.5% (organization B: 54.0% and C1: 81.4%).

### Study procedure

The participant flow and study procedure are presented in Fig. 1. First, before the intervention stage, we conducted the baseline survey. After the survey, we carried out interventions and observations of cognitive ergonomics and psychosocial stressors. This material is included in the qualitative baseline data, and is also used for tailoring the detailed content of the intervention material on the stratum level. We conducted randomization before the intervention stage.

The intervention stage consists of two parts. The first stage is a small-group workshop that initiates the intervention, and results in material to be used in tailoring the second stage of the intervention. The second stage is the implementation of the intervention, which includes intervention task reminder questionnaires.

Finally, after the intervention, the end-of-treatment survey, as well as two follow-up surveys are conducted. After the end-of-treatment survey, an evaluation workshop is organized to support the implementation during the follow-up phase and to provide qualitative data on the effects of the intervention. Other post-intervention actions include communication with the contact persons of the participating organizations.

### Materials

The study methods for collecting data were study questionnaires, an interview and observation method, an intervention workshop method, intervention task reminder questionnaires, and an evaluation workshop method, as described below.

#### Study questionnaires

The study consists of four assessment phases and related questionnaires: baseline (BL), end-of-treatment (EOT), follow-up at four months (FU04) and follow-up at ten months (FU10). Table 3 describes the nine questionnaire modules used.

**Table 3:** Study questionnaire modules, description of their content and their assessment schedule

Module	Description	BL	EOT	FU04	FU10
Background *	Nine items on the background of the participant, e.g. age, gender, education, and length of employment.	IGs, PC			
Working Conditions	Ten items on working conditions (developed at FIOH), e.g., number of hours, projects and places to work in a typical work week.	IGs			
Performance	Two items from the Health and Work Performance Questionnaire [39, 40].	IGs, PC	IGs, PC	IGs, PC	IGs, PC
Brain Work Profile (BWP)	BWP questionnaire (developed at FIOH) addresses cognitive demands of work with 39 items in 13 categories. The categories include working amidst disruptions and interruptions, as well as linguistic, memory, and multitasking demands, among others. Each item is assessed for both the frequency and the level of strain vs. engagement.	IGs, PC	IGs, PC	IGs, PC	IGs, PC
Knowledge Work Cognitive Failure Scale (KWCFS)	Modified version of the Workplace Cognitive Failure Scale (WCFS, [49]), with some knowledge work specific items added, resulting in 18 items on cognitive failures in knowledge work, with five subscales: Memory, Attention, Multitasking, Instructions, and Interruptions.	IGs, PC	IGs, PC	IGs, PC	IGs, PC
Copenhagen Psychosocial Questionnaire II (COPSOQ-II)	Seven scales with 23 items [41, 42]: Quantitative demands, Cognitive demands, Work-family conflict, Family-work conflict, General health perception, Burnout, and Cognitive stress.	IGs, PC**	IGs, PC**	IGs, PC**	IGs, PC**
Stress and Recovery	Two items assessed on a scale 0 (not problems at all) to 10 (severe problems)	IGs, PC	IGs, PC	IGs, PC	IGs, PC
Actions	Eighteen items that might affect workflow, such as decreasing the level of office noise or reducing the number of meetings, which were assessed to determine whether the action would improve or reduce workflow.	IGs			
Change	Six items assessed for the level of change, one for each treatment and active control intervention theme: Disruptions, Interruptions, Information Overload, Strain, Recovery, and Work/Life Balance.		IGs, PC	IGs, PC	IGs, PC

*BL* baseline, *EOT* end-of-treatment, *FU04* follow-up at four months, *FU10* follow-up at ten months

*IGs* Intervention groups, include both the Cognitive Ergonomics Intervention group (treatment) and the Recovery Support Intervention group (active control), *PC* Passive Control group

* The background module was included in the EOT survey for those participants who were added after the BL or had not responded the BL survey

** PC received only the Work-Family Conflict and Burnout scales from the COPSOQ-II

The questionnaires were conducted via a secure web-based questionnaire service. At the beginning of each questionnaire, the participants received an email invitation with a description of the study and the questionnaire and reasons to participate. They had about three weeks to answer the questionnaire. Those who had not answered the questionnaire received one to two reminders per week.

#### Interviews and observations

The aim of the semi-structured interviews and observations was to obtain detailed, qualitive information on the disruptions, interruptions, and information overload related to the work tasks of participants. Stress management, recovery, balance between work and leisure time, and the resources that support work performance were also covered. The focus of the interviews and the observations was to determine the participants’ general work characteristics (working time in terms of ICT, meetings, and other tasks) and typical tasks at work. We observed both challenges to and good solutions for performing cognitively demanding work tasks, as well as situations that affect cognitive strain, workload and recovery. Interviews and observations were conducted either at the participant’s desk or, if they worked in an open office, in a meeting room. Each session took about 1.5–2 h. Seven interviewers – psychologists who were trained in the use of the method (including VK, SS, and HJ, and one graduate student of psychology who was completing obligatory clinical training) – conducted the interviews and observations.

Before giving their written consent, the participants received both oral and written information on the study, interview, and observation. The semi-structured interview started with a few minutes of information on the contents of the interview, followed by a 20- to 30-min background interview on education and work experience, work tasks, and the demands and settings of work. The next stage included a 45- to 60-min observation of the participants’ typical work tasks. Participants had been instructed to think aloud and explain what they were doing. Depending on their work, we focused on 3–7 typical tasks, sometimes some more important common tasks. In the end, a short interview completed the information on the conditions related to strain, workload, engaging factors, and recovery, and enabled a discussion on anything the interviewee wished to add or ask. If the interviews and observations were conducted in a meeting room, a short visit to the participant’s office or desk was also included to obtain information about the actual work environment.

#### Cognitive ergonomics intervention at the workplace, CogErg

The intervention method, Cognitive Ergonomics Intervention Programme at the Workplace (CogErg), has been developed at FIOH in recent years in cooperation with various organizations and occupational health and organizational psychologists. The programme was refined and described in detail in the study intervention manual (version 2018-02-06, available in Finnish) for both the cognitive ergonomics intervention (treatment) and equivalent recovery support intervention (active control), before the start of the intervention. The intervention includes two steps: 1) the initial workshop, and 2) providing support during the implementation phase. The intervention is carried out in an authentic work context in co-operation with the employees, and the focus is on the work community level, that is, teams and other organizational groups working together.

The basic idea of CogErg is that the work community develops shared, good and concrete practices to improve cognitive ergonomics, and implements these practices in their daily work. The intervention thus aims to enhance group-level behaviour change, demonstrated as improved cognitive ergonomics in work and working conditions. The intervention applies the Mental Contrasting with Implementation Intentions (MCII) behaviour change method [44], utilizing its basic elements: first, the contrast between the current situation and future goals is demonstrated and, second, during the workshop and the implementation stage, concrete IF–THEN ground rules for work are defined. Furthermore, the workshop exercises and the reminders during the intervention implementation phase use the WOOP elements of the MCII method, that is, Wish, Outcome, Obstacle, and Plan [44].

#### The intervention workshop

The intervention starts with a workshop, organized either as one, two, or three equally long sessions, totalling three hours. The workshops are organized 4–8 weeks after the baseline survey, and the timeline is tailored for the participating organization. All participants are either present, or participate in the sessions via an electronic meeting system (e.g. video conference call). The treatment and active control groups participated in similarly structured workshops, but the specific content was different. The structure and content of the workshops are presented in Table 4.

**Table 4:** Outline of cognitive ergonomics and recovery support workshops

Phases	Description	Examples
Introduction of workshop	Presentation of workshop goals and information on the topics according to group allocation	Topics CE: 1. Disruptions, 2. Interruptions, and 3. Information overload.
		Topics RS: 1. Stress management, 2. Recovery, and 3. Work-life balance
MCII: Optimal situation vs results of baseline measurement	Optimal situation based on research evidence vs organization’s current situation according to questionnaire, interviews and observations.	Same structure for both intervention arms.
Informed consent	Oral and written information on the study, signed informed consent. If all participants agree, the workshop discussion during the second part is recorded	Same structure for both intervention arms.
Task assignment and groupwork (3–4 persons/group)	Planning practices to improve current situation under intervention arm topics. The group chooses a spokesperson as well as a record-keeper, who keeps the schedule and saves the group’s answers on a structured intervention sheet.	Same structure for both intervention arms.
WOOP method:		
Wishes (W) and Outcomes (O) (10 min)	Wishes and optimal outcomes are sketched for the group. Group discussion: i) importance of issue in the work community ii) indication whether issue is in order or resolvable and iii) other comments and questions	CE: “There isn’t too much noise, and speech is muted: there are no unnecessary distracting ringtones or movement (W); we have agreed on ground rules and working methods that reduce noise and distractions (O).”
		RS: “The strain factors are in balance with resources at work; the amount of strain at work is appropriate (W); We have agreed on ground rules and working methods that help us manage strain (O).”
Obstacles (O) (10 min)	Naming the concrete obstacles (e.g. situations) that prevent the achievement of outcomes and how to deal with them.	CE: Discussion and identification of distractions that hinder work and flow of tasks, such as speech, noise, and people passing
		RS: Discussion and identification of specific working conditions that increase strain, such as situations resulting in lunch breaks being skipped
Plan (P) (50 min)	Formulating ground rules (GR) and describing the working method by using IF-THEN rules, arguing why the issue is important (I) to the group, and discussing how the group can make the change (C) work together. Working with both ready-made examples and own rules describing the working methods.	CE: “We’ll control our voices”. IF I work in a space where there are other people, THEN I will lower my voice and keep the noise level low. (GR) “In many spaces noise is a major straining factor, every one of us can affect the noise level around us” (I). “Person X will bring the issue up in a team meeting” (C).
		RS: “Schedules are predictable”. IF unpredictable tasks arise often, THEN there is time reserved in the calendar for them. (GR) “Scheduling time for unpredictable tasks reduces haste and time pressure” (I). “Supervisor Y ensures that the matter moves forward” (C).
Utilization and dissemination (5–10 min)	Consideration of concrete implementation of behaviour change methods to ensure success. Structured evaluation i) of the usefulness of the workshop and ii) of the engagement in the behavioural change.	Concrete methods for both intervention arms; for example, what things should be further discussed and with whom; what message needs to be passed on, how and to whom; what long-term measures should be undertaken
Conclusion	Discussion and sending of files to research contact person.	Same structure for both intervention arms.

*MCII* Mental Contrasting Implementation Intentions, *WOOP* Elements of the MCII method, Wish Outcome, Obstacle, and Plan, *GR* Ground rule, *I* why the issue is important, *C* how the group can make the change, *CE* Cognitive Ergonomics group, *RS* Recovery Support group

The first part consists of an introduction, which describes the goals of the workshop and information on the three focus themes based on intervention group allocation (cognitive ergonomics, CE, or recovery support, RS), and the key results of the baseline survey and the interviews. The presentation material is then framed to reveal the mental contrast between an optimal situation and the current situation in the work community. We present the results by showing that the optimal situation based on research knowledge on workplace well-being and productivity is not yet realized in the work community.

The next part focuses on planning good, concrete practices that improve the current situation under the three main themes of the workshop. Participants work in groups and are provided with detailed instructions for discussion and a questionnaire that supports developing the work community’s common working methods under the intervention themes (CE or RS).

The workshop tasks follow the WOOP method of MCII. In the first tasks (about 10 min) the Wishes, that is, the three themes of the workshop, are named and the optimal outcomes are sketched for the group. The idea of this task is to enhance mental contrasting by realizing and discussing the current situation and any possible outcomes, and to increase commitment to the wishes and outcomes.

The second workshop task (about 10 min) focuses on obstacles, and the group is instructed to discuss concrete situations in which workflow suffers. The idea of this task is to help the group notice the obstacles to the outcomes. It is important to notice that our study focuses on the obstacles related to working conditions and working methods, rather than inner mental obstacles, which are the focus of other studies applying WOOP or MCII (see for example [44, 45].

The third workshop task (about 50 min) is about developing ground rules. The aim of this task is to develop concrete working methods that lead to outcomes, and to formulate them as organization-specific IF–THEN rules to enhance behaviour change. Two examples are given for each theme, and the group is asked to assess how suitable the named working method is for them. The examples are tailored to be relevant to the participating organization and are based on the material obtained from the baseline interviews and observations.

The fourth workshop task (about 5–10 min) for the group is to discuss and assess how they can ensure success and prepare to bring their view to the joint final discussion of the workshop. The idea of the fourth task is to increase commitment to and activity during future steps.

The workshop ends with a short joint discussion that brings together some ideas from different sub-groups that joined the workshop and provides information on the next steps of the study. The workshops are delivered by four psychologists and three specialist/researchers (including VK, HJ, TV) who are trained to present the material and lead the workshop.

#### Intervention task reminders

The implementation phase starts 2–12 weeks after the workshops in the organization and the time line is tailored for the participating organizations. All employees of the participating units receive an email once or twice a week containing short task questionnaires that take about five minutes to complete during working hours. Altogether 10 task reminder questionnaires are sent to the employees and supervisors, and three questionnaires are sent to the supervisors only. The task questionnaires remind participants of the issues agreed upon, ask how well the ground rules work in practice, and include some items related to presence and well-being at work (see Table 1). The purpose of these short questionnaires is to support the implementation of the new working methods and the ground rules agreed on during the workshops. The structure of these reminders is the same for the treatment and the active control group, but the content follows the themes relevant to each group. Furthermore, the good practices named in the workshop task replies of each organization are formulated to organization-specific IF–THEN rules, making also the examples and detailed content in the intervention task reminders organization-specific.

#### Evaluation workshop

Evaluation workshops will be arranged for each of four organizations between the end-of-treatment and follow-up surveys, and separately for the Cognitive ergonomics (treatment) and the Recovery support groups (active control). The evaluation method is interactive and learning-oriented, and combines the benefits of participatory and external evaluation by applying an aquarium method and a multi-criteria evaluation framework that supports multi-voiced evaluation (described in more detail in [46, 47)]. There are six dimensions for evaluating the impacts of the service innovation (in our study, the impact of the intervention): impacts on the citizen, the employee, and the population; and impacts on reputation, integration of technology and services, and economy.

The length of each evaluation workshop is 2–3 h. Participants sit in two circles and discuss and evaluate the success of the implementation of the intervention and new ways of working. The inner circle consists of 3–8 members who have participated in the intervention implementation phase, and they are asked to first discuss and evaluate the six dimensions of the intervention. The outer circle, with 3–6 members (e.g. managers, well-being/safety specialists, others who have not participated in the intervention) listen carefully and then comment on the discussion and suggest concrete actions for going forward.

### Tailoring

The detailed content of the CogErg intervention and the detailed timeline of the intervention’s implementation are tailored to suit the participating organizations. However, the three focus themes, the structure of the workshop and the structure of the implementation are constant and the same for all the participating organizations. In the workshop, the detailed content of information is tailored for the organization in question, that is, the baseline results of the survey and the interviews/observations, and the IF–THEN rules that are given as examples in the workshop tasks. Furthermore, the good practices named in the workshop task replies of each organization are formulated into organization-specific IF–THEN rules, making also the examples and detailed content of the intervention task reminders organization-specific.

The tailoring of the implementation of the measurements and intervention has the following components: whether the organization invites all or only a selection of employees to the workshop; whether the three-hour workshop is arranged as one, two or three sessions; and whether the workshop is arranged live or held via a technological communication system. Furthermore, organizational situations (such as busy periods, holiday periods, communication culture) may affect the exact timing of the intervention task reminders, as may as the number of reminders sent to participants that have not responded to the survey and questionnaires. Activities for communicating with the organization are tailored to their needs.

### Outcome measures

The outcome measures are based on the study questionnaires presented in Table 3. The items of the measures are presented in Table 2. The primary outcome measure of the study is Cognitive Strain Prevalence, the average of three subscales: Disruptions, Interruptions and Information Overload. The subscales are weighted averages of items from the Brain Work Profile and Knowledge Work Cognitive Failure Scale questionnaire modules. The participants are asked to “*Think of your work over the last month and for each question, mark how often your work required you to do the things presented*”. For example, “*Work in a noisy environment or with speech in the background?*” (Disruptions); “*Interrupt a task that is underway?*” (Interruptions); or “*Monitor several things and observe changes?*” (Information overload). The items are assessed for the frequency of dealing with the named cognitively demanding working conditions on a five-level scale: Multiple times per day, Daily, Weekly, Monthly, or More rarely. Responses are transformed into times-per-week values (10, 5, 1, 0.22 and 0 times per week, respectively) to approximate a continuous variable for calculating the averages. When counting the subscale averages at each study phase, the variables are weighted by their factor loadings in the baseline survey.

We also analyse the effect of the treatment on several secondary outcome measures, described in Table 2. The Subjective Cognitive Strain measure includes the same 15 items as the Cognitive Strain Prevalence measure, but the items assess subjective strain on a six-level scale: Energizing – a lot, Energizing – quite much, Energizing – a bit, neither energizing nor straining, straining – a bit, straining – quite much, straining – a lot. Second, we also use all 39 items from the Brain Work Profile questionnaire module as a measure of general Subjective Cognitive Strain. There are 13 cognitive categories, including the Cognitive Strain Prevalence items, but also items related to language processing, visual demands, problem-solving, and other cognitive demands. The question in this measure is “*You answered that your work requires you to do the following at least monthly. How do you feel about these things*? Do they motivate / energize you, or do they cause you strain?”. Examples of items included in this measure are “*Read or write instructions, messages or documents?*” (Language); “*Be able to see small visual details?*” (Visual); and “*Find alternative solutions?*” (Problem solving).

Well-being includes four measures. Subjective stress is measured with a stress-symptoms item that has shown satisfactory content, criterion, and construct validity for group-level analysis [48]. The experience of stress is assessed on a scale of 0 to 10: “*By stress we mean a situation in which a person feels tense, restless, nervous, or anxious, or they find it difficult to sleep because they cannot switch off their thoughts. Do you currently feel this kind of stress?”* A similar recovery item is assessed on a scale of 0 to 10: “*How well do you usually feel that you recover from the strain caused by your work (both mental and physical) after your working day/work shift?*”. Our General Health measure is a single item from the COPSOQ-II questionnaire [41, 42] and our Burnout measure the four-item COPSOQ-II Burn Out index (Cronbach alpha 0.83), including, for example, the item “*How often have you felt worn out?*“.

Cognitive Stress Symptoms (Cronbach alpha 0.83) is also a measure of the COPSOQ-II questionnaire [41, 42], including, for instance, the item “*How often have you had problems concentrating?*“.

Work Flow and Productivity measures include four measures. Presenteeism and Subjective Productivity measure includes “*How would you rate your own everyday performance during the last one or two years?*” [39]. The three Cognitive Failure measures are assessed for “*How often does the following happen to you in your work?*”, for example, “*You do not remember a password, numerical series, etc. that you need for your work*” (Memory Failures Measure); “*Your attention easily turns from your own work to your colleagues or what they are doing*” (Attention Failures Measure); and “*You find it difficult to decide which of several tasks is the most important to complete*” (Multitasking Failures Measure). Memory and Attention Failure measures are adapted from the Cognitive Failure Scale which shows high factorial, construct, and criterion-related validity [49].

## Plan of analysis

The analyses will include primary and secondary outcome analyses, post-hoc analyses, qualitative analyses, and process evaluation. The results will be reported in multiple articles dedicated to specific aspects of the study.

### Outcome analyses

The primary outcome variable is Cognitive Strain Prevalence (see Table 2). Descriptive statistics (frequencies, means and SD), the Mann-Whitney U-test, and chi-squared test will be used for baseline, end-of-treatment, and follow-up (4 month and 10 month) data to determine whether participant and group characteristics are comparable in the treatment and active control groups (randomized groups) and the passive control group (non-randomized group). The main longitudinal data analyses will be based on the Linear Mixed Modelling procedure, including incomplete cases in the analysis and assumes that missing data are random. The data are naturally hierarchical and consist of four organizations, organizational units (clusters), and individual respondents. Organization- and individual-level factors (e.g. gender and age) are included in the model, and the main level of the primary analyses will be that of the cluster. Item-level missing or invalid values are expected to be few, due to the computerized response format, and are not replaced.

Furthermore, additional analyses will include the non-randomized passive control group data in the baseline and end-of-treatment phases. Additional primary outcome analyses will also include post-hoc analyses with possible moderators in the model. The moderators will be defined on the basis of the process evaluation analyses.

Secondary outcome analyses will be the same as for primary outcome measure. We will also analyse subjective productivity measures using difference-in-difference regression. Additional post-hoc analyses such as mixed graphical models or trajectory analyses will also reveal, for example, the factors that predict successful change in a group.

### Process evaluation and qualitative analyses

Process evaluation is based on the quantitative and qualitative data obtained from the intervention workshop, intervention task questionnaires, and the evaluation workshop. The quantitative project evaluation variables will be related to the implementation of intervention, organizational context factors, and the mechanisms of impact, such as the reach of the intervention (e.g. response % within the cluster), the commitment of the organization and supervisors (e.g. response activity of the supervisor), and participant responses to the intervention (e.g. number of concrete examples reported by the cluster members), respectively. Based on these factors, we will categorize each group (cluster) by the success of the implementation. We will use the outcomes of the process evaluation as moderating factors in the secondary and additional analyses (see above).

Other qualitative analyses will be based on the open-item responses in the baseline, intervention task reminder, and end-of-treatment surveys. We will use qualitative content analyses to study how the discussed themes develop between the baseline survey and the end-of-treatment survey in the treatment and active control groups: what the main themes are, whether there are differences between the themes or their development in the studied groups, and what new and old themes rise or disappear in the different measurement phases.

The qualitative analyses of the workshops include the content analysis of discussions: we will classify the characteristics of interaction and activity in the groups, the ideas and solutions created, and the disturbances and problems. The voice-recorded and transcribed intervention and evaluation workshop discussions and observations, including field notes, will also be contrasted with open-item questionnaire responses in the baseline, end-of-treatment, and follow-up phases.

## Discussion

Today, cognitive demands of work life tasks are high, and they will remain so in the future. Although the cognitive strain related to work environments and ways of working is widely recognized and actively discussed as a notable risk factor, few studies have directly and systematically aimed to create conditions that provide better support for humans to perform cognitively demanding tasks (e.g. [19, 24, 31–34]). Knowledge of effects of cognitive strain on work performance and employee well-being, as well as intervention research on managing work conditions to support performance and well-being, needs to be expanded. This study applies the cognitive ergonomic workplace intervention, CogErg, to knowledge work in offices and examines how effectively it reduces the frequency of cognitively straining conditions as a primary outcome measure. Furthermore, several secondary outcome measures reveal its influence on subjective well-being and productivity. We use both quantitative and qualitative measures to evaluate the intervention process and to study the factors that moderate the effects of the intervention. To our knowledge, no previous studies have done this in such settings.

The strengths of this study are its longitudinal nature, RCT design with randomized organizational groups (clusters), and an active control group in which only the content of the intervention differs; the structure of the intervention is the same as that in the treatment group. Thus, the results will reveal if it is the specific cognitive ergonomics content of the intervention that contributes to the possible effects, that is, improving cognitive ergonomics of work conditions and ways of working (treatment) rather than supporting recovery (active control). Furthermore, as this study also includes a (non-randomized) passive control group, the results will provide a reference to a condition with no intervention actions (although the conclusions are limited due to the non-randomized nature of this group).

Furthermore, the study focuses on three common cognitive strain themes; disruptions, interruptions, and information overload, and will provide a systematic, detailed picture of how these conditions actualize in knowledge work in offices. Although previous studies have been conducted on each theme (e.g. [4, 8–15, 31–34]), the strength of our study is that it provides a more general view of the main sources of cognitive strain and the importance of each one, which may depend on the organizational context, working conditions, and types of knowledge work tasks. Although our study approaches these three cognitive strain themes systematically, the implementation of the intervention is based on tailoring the detailed initiatives and new ways of working. Thus, our study will provide an extensive and practically useful source for examples of good cognitive ergonomic practices at workplaces, and examples of recovery support at work, as developed in the active control groups.

The RCT design allows us to conclude whether the intervention programme is effective. However, real-world interventions are complex and process analysis methods are required to reveal the details of the implementation of the intervention and to recognize which actions and when, and where these actions are effective in real work life [36–38]. One strength of our study is that data are also collected during the intervention phase, and that we use quantitative and qualitative methods to assess the implementation phase and the outcomes of the intervention. The longitudinal follow-up provides a great deal of information during implementation, and combined with the RCT design, the process evaluation will reveal the factors underlying the effectiveness of the intervention. Combining the process evaluation results with the quantitative analyses for the effects on the primary and secondary variables allows us to understand which moderating factors affect whether or not the intervention is effective.

The main research question of our study is whether a cognitive ergonomics intervention can reduce the frequency of cognitive strain in working conditions. However, we will also analyse whether the intervention affects subjective performance and well-being. The results will also provide information on the effects of recovery support intervention and will clarify whether the outcomes of the intervention that focuses on reducing cognitive strain in working conditions and ways of working are different to those of the intervention that supports recovery. Thus, our study combines the research field that focuses on job design and job crafting for improving work performance and the research field of employee well-being and its consequences. The results will contribute to the current discussion on whether productivity and well-being at work have mutual connections, that is, the happy worker-productive worker thesis [19].

However, certain limitations deserve attention. Our study approaches groups of employees within the organization, and the intervention includes both individual and organizational level elements. The participative elements during the workshop and implementation aim to commit individual employees and supervisors and support behaviour change among individuals within the groups. On the other hand, the focus is on common working conditions, and related changes require organizational-level commitment and decisions. Thus, successful implementation would require actions on both the individual and organizational level. Selection bias within the groups may result if those who volunteer to participate in the study condition are more interested in the named themes than the non-participating employees. For example, those motivated to change working conditions may be overrepresented in the treatment group, whereas in the active control group, the participants motivated to enhance recovery may be overrepresented and more active during the implementation phase. This kind of selection bias could also increase if the basic level of the primary and secondary outcome variables differs between the participating individuals in the groups, despite randomization.

Moreover, as our complex workplace intervention study occurs in a naturalistic setting during a long intervention and follow-up period, many kinds of occupational and organizational changes are likely to occur. These may have an impact on both individual and organizational level participation, such as the motivation to remain in the study, or the prioritization between intervention implementation and development and other projects relevant for the organization. Although these individual and organizational level challenges may create limitations to the study, our approach nevertheless attempts to handle these problems. During implementation, we provide the individuals with support and actively communicate with the participating organizations, which receive useful information about their own situation throughout the study. Furthermore, process evaluation data and communication with our contact persons will allow us to recognize changes in the organizational context, and thus discuss the possible limitations of intervention implementation in detail.

To conclude, our study will provide new information on several key issues regarding cognitively demanding tasks, productivity, and well-being, which are highly relevant to modern work life. Our randomized controlled trial will reveal the possible beneficial effects of the cognitive ergonomics workplace intervention. The results will also suggest which evidence-based cognitive ergonomic practices best support work performance in cognitively demanding tasks. Since the intervention is implemented in actual workplaces, and the employees participate in the development of good practices for knowledge work, the project will provide many examples of concrete methods and ways in which to improve actual working conditions and well-being at work. Thus, the results will also provide practical implications for workplaces, human resource and occupational health experts, and decision-makers.

## Data Availability

Not applicable.

## Abbreviations

BL: baseline
BWP: Brain Work Profile questionnaire
CE: Cognitive ergonomics intervention
CogErg: Cognitive Ergonomics Intervention at the Workplace
COPSOQ-II: Copenhagen Psychosocial Questionnaire II
EOT: end-of-treatment
FIOH: Finnish Institute of Occupational Health
FU04: follow-up at four months
FU10: follow-up at ten months
GR: Ground rule
ICC: intraclass correlation coefficient
IGs: intervention groups, include both CE and RS
KWCFS: Knowledge Work Cognitive Failure Scale
MCII: Mental Contrasting with Implementation Intentions
MCQ: multiple choice question
OQ: optional question
PC: passive control group
RCT: randomized controlled trial
RS: Recovery support intervention
SQ: intervention task reminder questionnaires for supervisors
TQ: intervention task reminder questionnaires for employees
WOOP: elements of the MCII method, that is, Wish, Outcome, Obstacle, and Plan
